# Bisphosphonate’s Effect on Tongue Mucosa: An Experimental Electron Microscopy Study

**DOI:** 10.3390/medicina56020051

**Published:** 2020-01-24

**Authors:** Theodora Papamitsou, Antonios Morsi-Yeroyannis, Anastasios Papanastasiou, Nikolaos Bakalopoulos, Eva-Maria Dietrich, Sofia Karachrysafi, Alexandros Toskas, Evangelia Mareti, Anastasia Morsi-Yeroyanni, Antonia Sioga

**Affiliations:** 1 Laboratory of Histology-Embryology, Faculty of Medicine, School of Health Sciences, Aristotle University of Thessaloniki, 54124 Thessaloniki, Greece; thpapami@auth.gr (T.P.); alextoskas@hotmail.com (A.T.); 2 Faculty of Medicine, School of Health Sciences, Aristotle University of Thessaloniki, 54124 Thessaloniki, Greece; amorsige@gapps.auth.gr (A.M.-Y.); tasos-papana@hotmail.com (A.P.); bakalopoulosnikos@gmail.com (N.B.); anastasia.morsiyeroyanni@gmail.com (A.M.-Y.); 3 Department of Oral and Maxillofacial Surgery, University Hospital of Erlangen, 91054 Erlangen, Germany; aeffchen.dietrich@gmail.com; 4 Department of Ophthalmology of General Hospital G. Papanikolaou, 57010 Thessaloniki, Greece; sofaki.kara11@hotmail.com; 5 2nd Department of Obstretics and Gynecology, General Hospital Hippokration, Aristotle University of Thessaloniki, 54642 Thessaloniki, Greece; eva-med@hotmail.com

**Keywords:** biphosphonates, tongue mucosa, electron microscopy

## Abstract

Background and objectives: Bisphosphonates (BPs) are selective inhibitors of osteoclasts, used for the treatment of bone disorders. The objective of this study is to investigate the possible effects of BPs on the tongue’s mucosa. Materials and Methods: Specimens of the tongue of 20 female 12-month old Wistar rats were taken. Ten were used as control group, while in the remaining alendronate (Fosamax, Merck) was administered per os from 13 weeks. Observation of the harvested samples was made by Transmission Electron Microscopy (TEM). Results: In the experimental group, focal alterations were observed to various extent in all specimens. The basement membrane was intact. Furthermore, an increase at the intercellular space was observed, predominantly at the middle layer, and the desmosomes were disorganized. In the lamina propria focal edema was observed. Conclusions: Investigation on the effect of BPs on the tongue’s mucosa through TEM hasn’t been documented in the past. According to our results, BPs seem to cause mild mucosal lesions on the tongue.

## 1. Introduction

Osteoporosis is commonly found in women, among other bone disorders. Its main characteristics are a decrease in bone mass and deterioration in the bone microarchitecture, that leads to more fragile bones. As a result, spontaneous or after minimal trauma bone fractures, are usual clinical symptoms of osteoporosis [1].

The discovery of bisphosphonates (BPs) was a real breakthrough for the treatment of osteoporosis. BPs (P-O-P) are non-hydrolysable analogues of inorganic pyrophosphate (P-C-P) in which a carbon exists instead of the bridging oxygen [2,3]. The main advantage of BPs is that they act selectively in the osteoclasts by binding to hydroxyapatite and inhibiting bone resorption [3,4]. Limited bone resorption by inhibiting adenosine triphosphate (ATP)-dependent enzymes is achieved by the first-generation of BPs, which results from their intracellularly metabolization to ATP analogues. On the other hand, the nitrogen-containing BPs act by inhibiting an essential enzyme of the mevalonate pathway, the farnesyl diphosphate (FPP) synthase [2,5,6].

However, BPs have many adverse effects. The most important of them concern the gastrointestinal tract including abdominal pain, dyspepsia, nausea, vomiting, constipation, diarrhea, bleeding, esophagitis, esophageal ulceration, Barrett’s esophagus, esophageal adenocarcinoma, gastric ulcer, cancer of gastrointestinal tract, oral ulcers and hepatotoxicity [7,8,9,10,11,12,13]. Moreover BPs can cause osteonecrosis of the jaw, musculoskeletal pain, hypocalcaemia and secondary hyperparathyroidism [7,14,15]. Finally, BPs have been associated with degenerative changes to nerves, like the inferior alveolar nerve as mentioned by Dietrich et al. [16].

It is generally known that BPs are not significantly absorbed by the gastrointestinal tract because of their poor lipophilicity. The oral bioavailability is very low (0.9–1.8%), and as a result, the concentration of BPs in the plasma is insignificant. Albumin is the predominant protein that binds BPs in the plasma, with pH and calcium concentration regulating the extent of their binding (70–80%). The not bonded BPs are cleared rapidly from plasma by deposition in bones and urinary excretion. After 24 h the remaining amount of the BPs lay in the bone tissues, from where they are gradually released, back into the circulation to be completely excreted by the urinary system. This procedure is very slow and can last up to 12 years in humans. Also, dispositing of the BPs in soft tissues (liver, kidney and spleen) is possible and depends on route and rate of drug administration and the osmolality of the vehicle [1,17,18].

This specific study tries to clarify the correlation between the BPs and their effects on the tongue. More specifically, and regarded as one of the side effects of BPs to the mucosa of the gastrointestinal system, a lot of studies have tried to connect the use of these drugs with damage to the mucosa of the tongue. There are also a lot of cases where patients, using bisphosphonates came up with ulcers or others forms of changes of the tongue like the loss of taste [3,19,20,21,22].

## 2. Materials and Methods

Approval by the Bioethics Committee of the Medical School of the Aristotle University of Thessaloniki was acquired for this study on 8th November 2012 with ID: 1.135/8-11-12. In the experiment, twenty female Wistar rats were used. They were 12-months-old and their weight was approximately 500 g. Each one of them was placed in different stainless steel cages with 12 h light-dark cycles, relative humidity and temperature control.

The animals were randomly allocated into one of two groups, group A (experimental) and B (control). Both consisted of 10 animals. Alendronate (Fosamax, Merck) at a dose of 0.05 mg/kg body weight/week dissolved in 3 cc normal saline for 13 weeks, was administered per os to the group A animals. The administration of the drug was performed thirty minutes prior to breakfast once a week. Calculation of the dose was made according to the human dose [14,23]. The study’s duration was limited to 13 weeks and after euthanasia, the tongue of the animals was removed, and specimens were prepared for electron microscopy examination. The specimens were randomly selected from the upper side of the tongue, since there was no macroscopic alteration on the tongue’s mucosa.

### Transmission Electron Microscopy (TEM)

Sectioning into <1 mm^3^ pieces was applied to the randomly selected tongue tissue samples which were then submerged into glutaraldehyde 3% for 2 h, followed by 1 h in osmium tetroxide (OsO_4_) 1%. Staining with uranyl acetate 1% for 16 h and dehydration with high ethanol concentrations followed. Finally, the specimens were embedded into Epon resin, ultra-thin sections (60–90 nm) were taken, which were stained with Reynold’s stain. Lastly, the tongue’s mucosa samples were examined in a TEM JEOL 1011 at 80 kV.

## 3. Results

**Figure 1:**
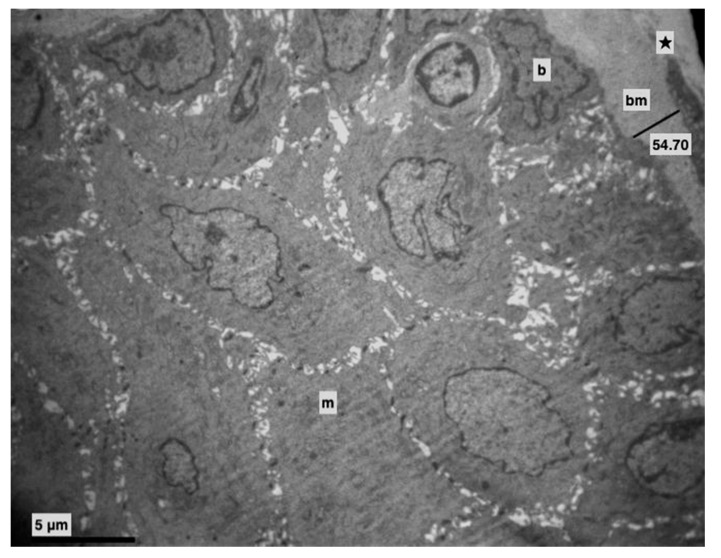
Control group. Basement membrane (bm) (Morphometric analysis: 54.70). Middle (m) and basal (b) layer. Lamina propria (★). (×4000).

All rats appeared to be in good health during the 13 weeks. In the control group alterations weren’t observed (Figure 1). No macroscopic changes were observed on the tongue mucosa either. 

In the experimental group, focal alterations were observed, in greater or lesser extent, in all specimens.

**Figure 2:**
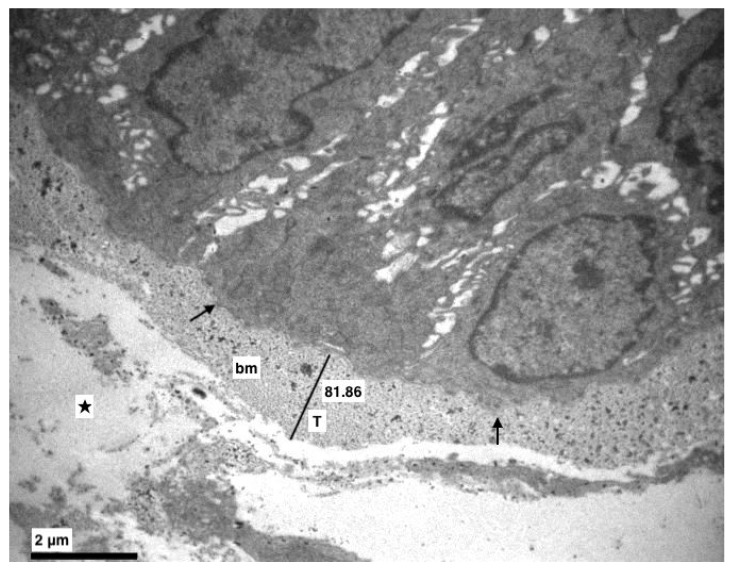
Experimental group. The basement membrane (bm) was intact but in some points thicker (T) (Morphometric analysis: 81.86). Dilution in the lamina propria (★), hemidesmosomes (↑). (×8000).

**Figure 3:**
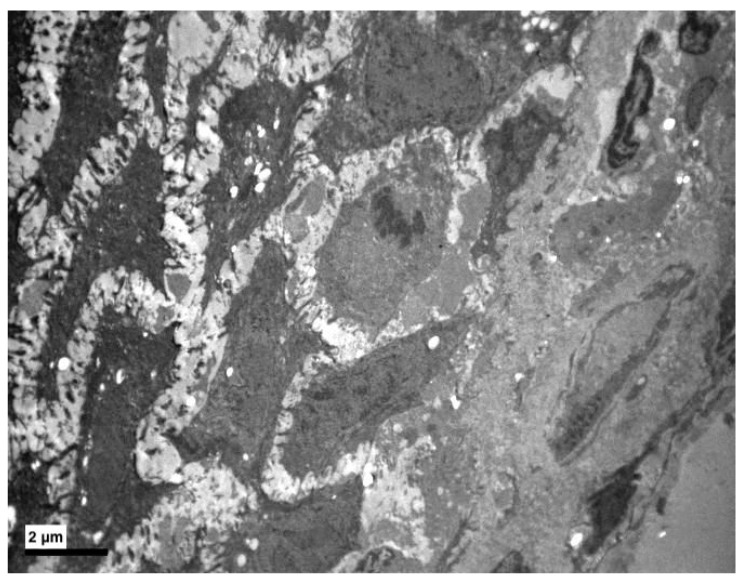
Experimental group. Divisions of basal cells near the basement membrane. (×2500).

More specifically, the basement membrane was always intact, sometimes thicker (Figure 1 and Figure 2), and the basal cells, where divisions were observed, were connected with hemidesmosomes (Figure 2 and Figure 3).

**Figure 4:**
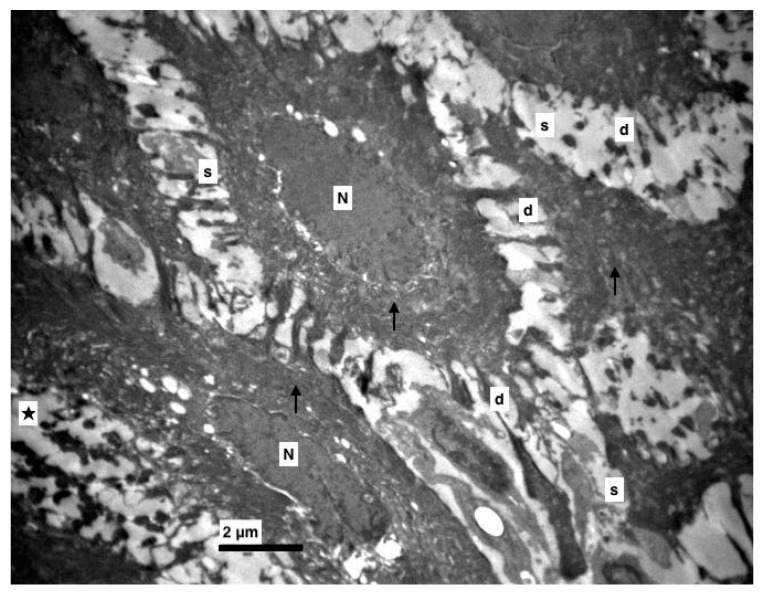
Experimental group. Middle layer of epithelium with increased intercellular spaces (s), destroyed (d) and accumulated desmosomes (★), nucleus of epithelial cells (N), thick bundles of tonofilaments (↑). (×5000).

**Figure 5:**
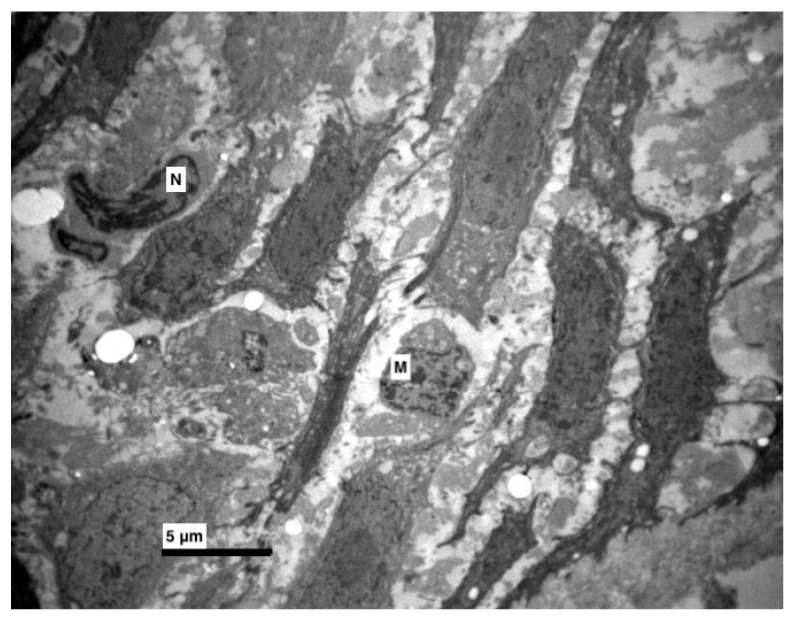
Experimental group. Increased intercellular spaces, with destructions of connections (desmosomes). Between epithelial cells macrophages (M) and cells with pyknotic nucleus (N) were observed. (×4000).

**Figure 6:**
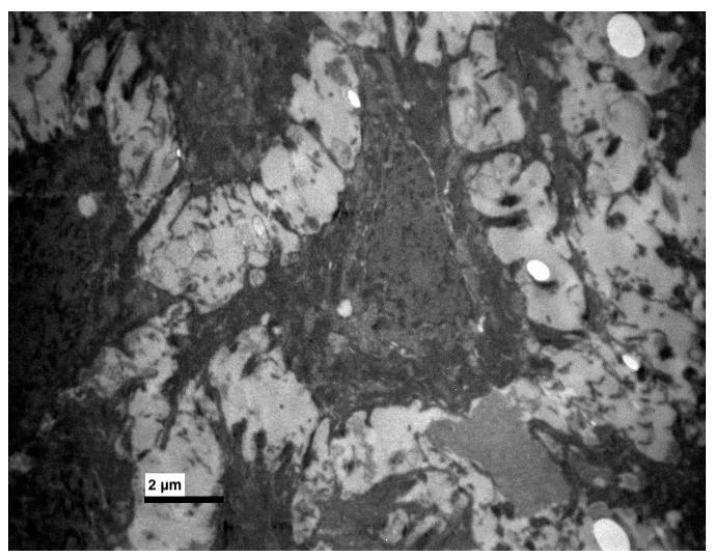
Experimental group. Thick bundles of tonofilaments and destroyed desmosomes. (×8000).

**Figure 7:**
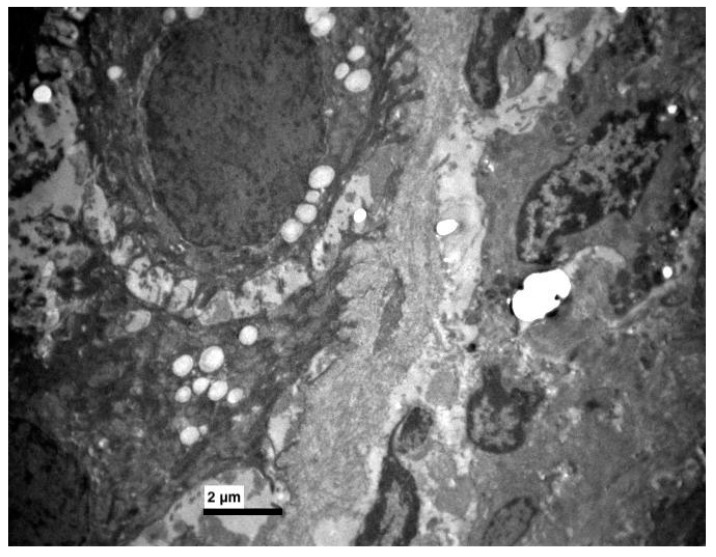
Experimental group. Few keratohyaline granules in some epithelial cells. (×5000).

**Figure 8:**
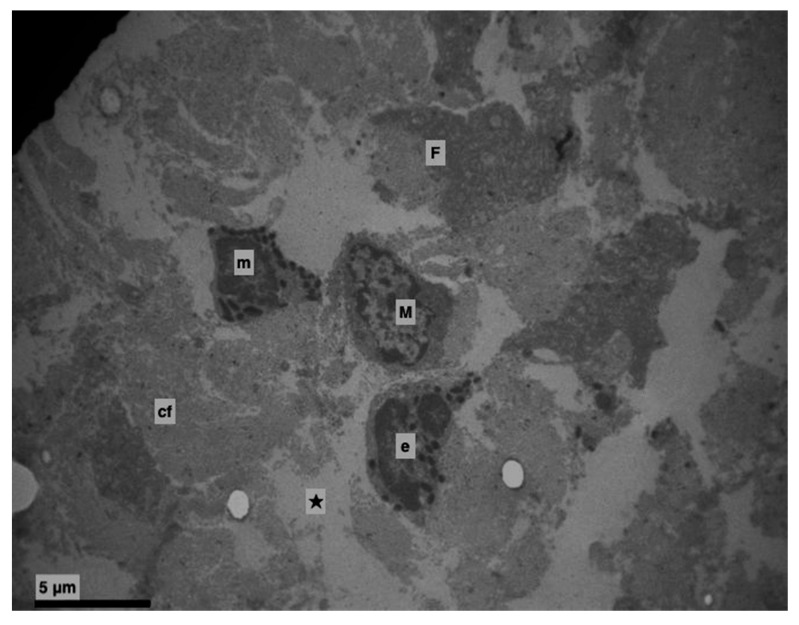
Experimental group. Lamina propria (★). Edema with destruction of collagen fibres. Eosinophil (e), mast cell (m), macrophage cell (M), active fibroblast (F), collagen fibres (cf). (×4000).

Furthermore, an increase at the intercellular spaces (ranging from being small to being very noticeable) was observed, predominantly at the middle layer and rarely at the basal. The desmosomes were between thin cytoplasmic processes (Figure 4, Figure 5 and Figure 6). Keratohyalin granules were very few and many thick bundles of tonofilaments were apparent in the middle layer (Figure 6 and Figure 7). Sometimes, lymphocytes and macrophages were found between the epithelial cells (Figure 5 and Figure 8).

In the lamina propria, focal edema, caused destruction of the collagen fibers and vividly active fibroblasts were observed (Figure 2 and Figure 8). Apoptotic cells, neutrophils, eosinophils, mast cells and macrophages as well were found (Figure 8).

## 4. Discussion

BPs are used for the treatment of bone disorders as well as for tumors for many years now and are selective inhibitors of osteoclast mediated bone resorption. Their adverse effects have been studied and reported in many previous studies and case reports. Some of them concern oral ulcerations. This study attempts to elucidate the possible effects of BPs on tongue cell structure and eventually in tongue function as there were no previous experimental studies to our knowledge about the side effects of BPs in the tongue’s mucosa.

Oliveira et al. noticed an increase of secretion granules in the cytoplasm of parotid and submandibular glands in rats under alendronate, either they were stimulated with pilocarpine or not. Moreover, biochemical tests identified an increase of total protein content and a decrease in amylase levels of the salivary glands of the pilocarpine-stimulated group, compared to the control group. Consequently, alendronate affects the structure and the function of the major salivary glands [24]. The saliva plays a protective role in the tongue.

Cruz et al. used gastro-resistant sodium alendronate-loaded microparticles prepared by spray-drying, which presented high encapsulation efficiencies. Specifically, their experiments showed good gastro-resistance in a low pH (1.2), while at a higher pH (6.8), the drug release was retarded. As a result, protection against gastric ulcer was feasible, that may lead to fewer side effects to the tongue [25].

In various case reports, the incorrect per os administration of alendronate has been associated with oral ulcerations, commonly on the palate or the tongue. After thorough examination extensive lesions, either bullous or ulcers, were described on the tongue. The healing of the oral ulcers, was achieved after the cessation or correct administration of the per os osteoporosis treatment, after just a few days, up until many months later [21,26]. Interestingly though, Kharazmi et al. (2010) presented a case of severe oral ulceration caused by inappropriate therapeutic administration of alendronate [27].

Additionally, Kharazmi et al. (2012) reported a review of the adverse effects of alendronate in oral mucosa. According to this, epithelium ulcers or necrosis was observed, accompanied by a subepithelial and/or perivascular lymphoplasmacytic infiltrate [21]. However, the main reason for this, is that alendronate acted directly on the tissues and there was prolonged local mucosal exposure because of misuse.

Donetti et al. (2014) performed biopsies of the oral mucosa of female patients, undergoing long-term oral therapy with alendronate. They observed, by immunofluorescence, a cutback in keratinocyte proliferation and a reduction in desmoglein 1 and keratin 10 expressions, and by ultrastructural studies, they observed that keratin filaments progressively condensed in the upper layers of the epithelium. In addition, in spinous and granular layers, aggregates of tonofilaments were present in the keratinocytes cytosol, while in the granular layer, nucleus abnormalities were detected. The keratinocytes were mostly found in the basal monolayer and their reproduction rate was decreased. Hemidesmosomes in the basal layer of the epithelium were found as well, but with changed morphology from the middle spinous layer, having a thinner desmosomal plaque. Intercellular spaces were normal. Lastly TEM and activated caspase-3 immunolabelling didn’t provide evidence of apoptotic induction in the oral mucosa. These findings are consistent with most of our results [22].

## 5. Conclusions

Taking everything into consideration, alendronate in therapeutic doses administered to clinically healthy rats, affects the structure of their tongues, and subsequently its function, among other affects. Moreover, according to the findings of the ultrastructural analysis, it could be argued that BPs may affect the human tongue’s structure too, even with correct per os administration. However, further research is necessary in order to have more reliable results about the negative effects of BPs in the structure and function of the human tongue.
